# Tumor cell cytoplasmic metallothionein expression associates with differential tumor immunogenicity and prognostic outcome in high-grade serous ovarian carcinoma

**DOI:** 10.3389/fonc.2023.1252700

**Published:** 2023-11-08

**Authors:** Elena Mairinger, Michael Wessolly, Paul Buderath, Sabrina Borchert, Larissa Henrich, Pawel Mach, Julia Steinborn, Rainer Kimming, Bharat Jasani, Kurt Werner Schmid, Agnes Bankfalvi, Fabian Dominik Mairinger

**Affiliations:** ^1^ Institute of Pathology, University Hospital Essen, Essen, Germany; ^2^ Department of Gynecology and Obstetrics, University Hospital Essen, Essen, Germany; ^3^ Department of Pathology, Targos - A Discovery Life Sciences Company, Kassel, Germany

**Keywords:** high-grade serous ovarian carcinoma, metallothionein, overall survival, immune response, zinc signaling

## Abstract

**Background:**

The underlying mechanism of high T-cell presence as a favorable prognostic factor in high-grade serous ovarian carcinoma (HGSOC) is not yet understood. In addition to immune cells, various cofactors are essential for immune processes. One of those are metallothioneins (MTs), metal-binding proteins comprising various isoforms. MTs play a role in tumor development and drug resistance. Moreover, MTs influence inflammatory processes by regulating zinc homeostasis. In particular, T-cell function and polarization are particularly susceptible to changes in zinc status. The aim of the present study was to investigate a possible role of MT-mediated immune response and its association with prognostic outcome in ovarian cancer.

**Methods:**

A retrospective study was conducted on a clinically well-characterized cohort of 24 patients with HGSOC treated at the University Hospital of Essen. Gene expression patterns for anti-cancer immunogenicity-related targets were performed using the NanoString nCounter platform for digital gene expression analysis with the appurtenant PanCancer Immune Profiling panel, consisting of 770 targets and 30 reference genes. Tumor-associated immunohistochemical MT protein expression was evaluated using a semi-quantitative four-tier Immunohistochemistry (IHC) scoring.

**Results:**

MT immunoexpression was detected in 43% (10/23) of all HGSOC samples. MT immunoexpression levels showed a significant association to survival, leading to prolonged progression-free and overall survival in positively stained tumors. Furthermore, T-cell receptor signaling gene signature showed a strong activation in MT-positive tumors. Activated downstream signaling cascades resulting in elevated interferon-gamma expression with a shift in the balance between T helper cells (T_H_1 and T_H_2) could be observed in the MT-positive subgroup. In addition, a higher expression pattern of perforin and several granzymes could be detected, overall suggestive of acute, targeted anti-cancer immune response in MT-positive samples.

**Conclusion:**

This is the first study combining broad, digital mRNA screening of anti-tumor immune response–associated genes and their relation to MT-I/II in ovarian cancer. MT overexpression is associated with molecular characteristics of an anti-cancer immune response and is a strong prognostic marker in ovarian HGSOC. The observed immune cell activation associated with tumor MT expression comprises but is not limited to T cells and natural killer cells.

## 1 Introduction

As a group, epithelial ovarian cancers (EOCs) are considered as perhaps the most aggressive malignancy among gynecological tumors worldwide. Although not as prevalent (n = 313,959) as breast (2,261,419), cervix (604,127), or uterine cancer (417,367), malignant tumors of the ovary still account for as much as 207,252 annually cancer-related deaths (1). Because of the absence of early symptoms, the majority of patients are diagnosed at an advanced stage. High-grade serous ovarian carcinoma (HGSOC), the predominant ovarian cancer subtype, is conventionally treated by radical cytoreductive surgery and platinum-based chemotherapy. However, the relative 5-year survival rate remains discouraging as the majority of patients will inevitably relapse (2).

Platinum analogs are genotoxic compounds inducing DNA damage and, subsequently, TP53-mediated cell cycle arrest and apoptosis (3). However, the reasons for impaired platinum therapy response remain uncertain (4–11), although different mechanisms causing it have been identified. These include (among others) reduced intracellular cisplatin accumulation due to altered transmembrane transport capabilities, activation of DNA damage repair pathways, activation of cell growth and cell fitness, aberrant DNA methylation, enhanced epithelial-to-mesenchymal transition, and reduced endocytosis of cisplatin (7–9, 12–15).

Metallothionein (MT) is a family of cytoplasmic, cysteine-rich, low–molecular mass proteins with heavy-metal–binding affinity due to the thiol groups of their cysteine residues (16). Four main isoforms have been shown to be expressed in humans (MT-I to MT-IV) with MT-I and MT-II representing the most prominent isoforms. Because of their role in multiple cellular processes, they have been shown to be expressed in various tumor entities (17). These processes encompass the regulation of oxidative stress, such as scavenging and inhibiting reactive oxygen species (17). Furthermore, a reduced cytotoxicity mediated by nitrogen could have been shown, in MT-overexpressing transgenic mice (18). MTs were reported as potential negative regulators of apoptosis and may play an important role in carcinogenesis and drug resistance (17). In addition, they mediate cellular resistance against various toxic heavy metals (19).

Furthermore, intracellular zinc homeostasis is mainly regulated by MTs (20). They influence inter-/intra-zinc finger cluster exchange events by their thiol groups (17, 21, 22). Zinc cation (Zn^2+^) is involved in catalysis or structural stabilization of over 300 enzymes. In addition, it is a component of an immense number of metalloproteases (20). Many of these are present in immune cells, making a comprehensive summary of all immune-related processes depending on zinc difficult to analyze (23). *In vivo*, zinc deficiency has been shown to alter the number and function of various immune cells, such as neutrophil granulocytes, monocytes, natural killer (NK) cells, T cells, and B cells (23). In particular, T-cell function and differentiation are susceptible to changes in zinc levels (23). However, not only zinc deficiency but also excessive zinc exposure has adverse effects on the immune system, leading to an inhibition of T-cell functions and an inhibition in the mixed lymphocyte reaction *in vitro* (24).

Different associations between MTs and immune infiltration could be observed. MT-I/II^−/−^ knockout mice show an increased number of inflammatory cells such as macrophages and T cells after injury, compared with the wild-type mice (25–27). Notably, MT-I/II have been shown to affect immune system processes such as immunoglobulin production (22, 27). Furthermore, Emri and colleagues presented a correlation among MT expression and the probability of metastasis in malignant melanoma (28).

The central role of the immune system in response to platin-based chemotherapy in various cancers has been intensely studied within the last decades. The absence of tumor-infiltrating lymphocytes (TILs) could be shown to be an independent predictor for platinum resistance (29). Moreover, platinum-resistant EOC could possibly be considered as a non-immune response cancer type, as the anti-tumor immune response, measured by the amount of TILs, is often downregulated (30). Similarly, increased CD3+, CD8+, and programmed cell death protein 1+ (PD1+) TILs can be found after neo-adjuvant platinum treatment in patients with EOC (31). In conclusion, a possible correlation between the mechanisms of platinum resistance and MT-associated immunodeficiency may be hypothesized.

However, the connection between clinical outcome and the patients’ anti-tumor immune-reaction as prognostic factors in HGSOC is still not understood. The aim of the present study was to obtain a deeper insight into the mechanisms of MT-mediated immune response in HGSOC.

## 2 Materials and methods

### 2.1 Demographic data

**Figure 1:**
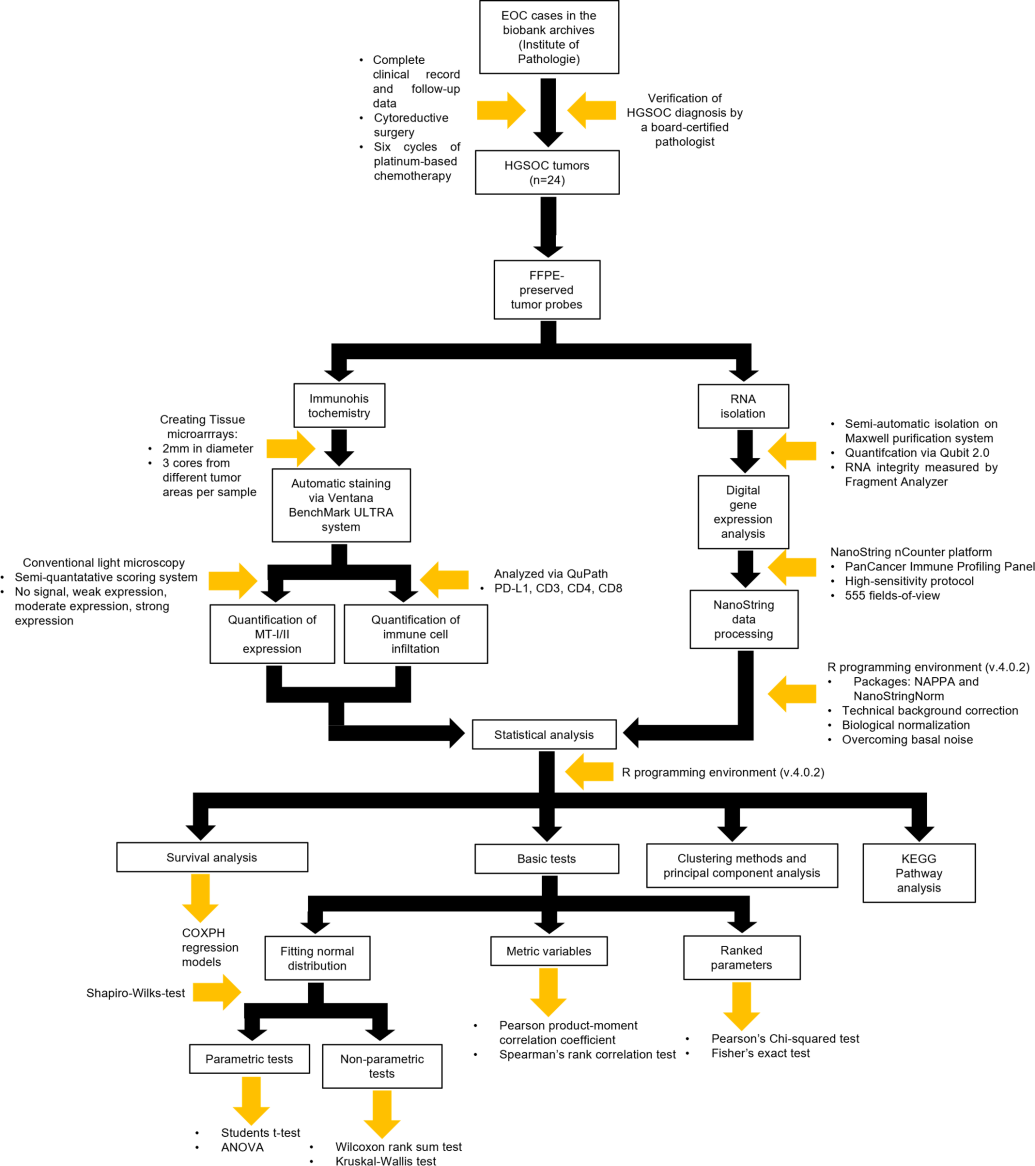
Flowchart encompassing the methodology of the entire project including study design, immunohistochemistry, digital gene expression analysis, and statistical analysis.

To investigate the potential influence of MTs on the immune response in EOC, 24 tumor samples were taken from a previously well-established patient cohort (32, 33). An overview of the working procedures of the entire study is given in **Figure 1**. Furthermore, the study was conducted retrospectively.

All tumor samples were collected at the Institute of Pathology (University Hospital Essen, Germany), where they were routinely deposited in the form of formalin-fixed, paraffin-embedded (FFPE) tumor blocks. An experienced pathologist (AB) reviewed and re-evaluated the initial diagnoses. Re-evaluation was performed on tissue sections stained with hematoxylin and eosin. All tumor samples were confirmed as HGSOC.

Treatment of patients was conducted at the University Hospital of Essen within a span of 5 years (2005–2010). Primary treatment consisted of cytoreductive surgery, followed by six cycles of adjuvant platinum-based chemotherapy. **Table 1** displays the general patient and tumor characteristics in the study cohort. Patients’ age at diagnosis ranged from 23 to 84 years, resulting in a mean age of 62 years across the cohort [standard deviation (SD) = 13.08]. Disease-free survival (DFS) varied between 4 months and 80 months, whereas overall survival (OS) fluctuated between 11 months and 126 months. Mean DFS and mean OS were 20.26 months (SD = 19.99 months) and 46.17 months (SD = 32.68), respectively. The observation period ended in October 2016. By then, 19 of the 24 patients had died (79.1%). The tumor recurrence rate among patients was rather high (83.3%).

**Table 1:** Patients characteristics.

Patients’ characteristics					
	n	%		n	%
Age			Relapse		
<65 years	12	50.0	Yes	20	83.3
≥65 years	12	50.0	No	4	16.7
Tumor size			DFS (months)		
<10 mm	2	8.3	Min	4.0	–
10–50 mm	13	54.2	Max	80.0	–
>50 mm	6	25.0	Median	9.0	–
NA	3	12.5	Mean	20.3	–
T-status			OS (months)		
1	1	4.2	Min	11.0	–
2	3	12.5	Max	138.0	–
3	19	79.2	Median	39.0	–
NA	1	4.2	Mean	48.7	–
N-status			MT localization		
0	6	25.0	Nuclear	1	4.2
1	11	45.8	Cytoplasmatic	7	29.2
NA	7	29.2	Nuclear + cytoplasmatic	2	8.3
			absent	14	58.3
M-status			MT expression binarized		
1	4	16.7	High	10	41.7
0	20	83.3	Low	13	54.2
			NA	1	4.2

NA, not assessed.

### 2.2 RNA extraction and digital gene expression analysis

The procedure of RNA extraction, integrity analysis, and digital quantification of mRNA expression has already been described in a previous study (33).

FFPE samples were utilized for RNA isolation (two sections of 10-µm thickness each). Isolation procedures were performed semi-automatically on the Maxwell purification system (Maxwell RSC RNA FFPE Kit, AS1440, Promega) as specified by the manufacturer. RNA was eluted in 50 µl of ribonuclease (RNAse)-free water and quantified using RNA broad-range assay on a Qubit 2.0 fluorometer (Life Technology). Finally, a fragment analyzer (Advanced Analytical Inc., Ames, IA, USA) was used to evaluate RNA integrity. For this purpose, the DNF-489 standard sensitivity RNA analysis kit was utilized.

Digital gene expression analysis was performed on the NanoString nCounter platform, using the dedicated PanCancer Immune Profiling panel. The panel consisted of 30 reference and 770 target genes associated with various immune processes and pathways. Minimum sample input was 100 ng of RNA for each reaction. Probes were hybridized on the nCounter Prep-Station (MAX/FLEX System) using the high-sensitivity protocol. Afterward, the cartridge was scanned on the Digital Analyzer (NanoString) at 555 fields of view.

### 2.3 NanoString data processing

NanoString data processing, the subsequent data analysis, and statistical analyses were performed in R (v4.0.2). Data were processed utilizing the NAPPA and NanoStringNorm package (34).

In the beginning, a technical normalization was performed in a two-tier process. First, the mean counts of the panel inherent negative controls plus two times the standard deviation were subtracted from all counts as technical background correction. Inherent positive controls, presented as dilutional series of artificial oligonucleotides, were used for determination of the dynamic range. Subsequently, a biological normalization using a normalization factor calculated from the geometric means of the included reference genes was performed. Actual expression signals were differentiated from basal noise by applying a one-sided t-test between the expression of the inherent negative controls and each gene. A p-value below 0.05 was considered as an actual signal.

### 2.4 Immunohistochemical staining

Systematic immunohistochemical analysis was performed on tissue micro-arrays. Blocks were cut into slices with a thickness of 4 µm. To take intratumoral heterogeneity into consideration, three distinct tissue cores (2 mm in diameter) from different tumor areas were used. Tissue staining was done automatically on the Ventana BenchMark ULTRA system (Ventana Medical Systems, Arizona, USA). Staining was performed according to the standard protocols of the manufacturer. Regarding the validation process, pleura samples served as negative control, whereas myoepithelial cells deriving from healthy breast tissue were used as reference tissue. MT-I and MT-II were stained using the clone E9 (Dako/Agilent, Santa Clara, CA, USA) as the primary antibody, because the targeted epitope is shared between the two MT isoforms (35). Samples were pretreated with a sodium citrate buffer for the purpose of antigen retrieval (0.01 M, pH 6). During pretreatment, samples were placed in hot water (90°C) with antigen retrieval solution for 30 min. The primary antibody was incubated at room temperature with a dilution factor of 100 and incubated for 30 min. For secondary (horseradish peroxidase (HRP)-conjugated) antibody application, the DAKO EnVision TM+ Visualization System, Peroxidase kit (Dako Cytomation, Cat. No. K4004) was utilized according to the manufacturer’s instructions.

A conventional light microscope (Nikon Eclipse 80i, Nikon Ltd. Düsseldorf, Germany) was used for evaluation of MT-I/MT-II expression by a board-certified pathologist. MT immune expression was quantified by a semi-quantitative scoring system. It consisted of four tiers based on the percentage of positively stained tumor cells, either in the nucleus or in the cytoplasm. The staining intensity was not taken into consideration. The cutoffs for the four tiers were expression in 0%, 1%–5%, 6%–50%, and, finally, more than 50% of tumor cells. The tiers were named as Score 0 (no signal), Score 1 (weak expression), Score 2 (moderate expression), and Score 3 (strong expression), respectively (35, 36).

In addition, slides were stained using antibodies against programmed death ligand 1 (PD-L1), CD3, CD4, and CD8 to analyze immune cell infiltration. Tissue samples were deparaffinized using xylene. For antigen retrieval, slides were incubated in cell conditioning solution 1 (CC1)/ethylenediaminetetraacetic acid buffer for 32 min, 40 min, 32 min, and 40 min, respectively. The samples were then incubated with the primary antibody for 32 min, 24 min, 32 min, and 24 min at room temperature. The dilution factors were 100, 200, 100, and 150 for the respective targets. Staining was performed according to the standard procedures as described above. The OptiView 3,3′-diaminobenzidine detection kit (760-700, Roche) was used for visualization. Slides were scanned using an Aperio ScanScope AT2 platform (Leica biosystems, Wetzlar, Germany) and analyzed with QuPath (v.0.2.0-m2, qupath.github.io/) as cells/mm². A list of all used antibodies and conditions is given in **Table 2**.

**Table 2:** Characteristics of antibodies used for immunohistochemical staining.

Marker	Company	Clone	Pretreatment (min)	Incubation (min)	Dilution
**PD-L1**	Roche	22C3	32	32	1:100
**CD3**	DCS	SP7	40	24	1:200
**CD4**	Zytomed	SP35	32	32	1:100
**CD8**	Dako	C8/144B	40	24	1:150
**MT-I/II**	Dako	E9	30	30	1:100

### 2.5 Statistical analysis

As described in Section 2.3, graphical and statistical analyses were performed in R (v.4.0.2).

Whether data resembled normal distribution patterns was examined by applying Shapiro–Wilks test. Rejection or acceptance of the null hypothesis resulted in applying a non-parametric (Wilcoxon rank sum test and Kruskal–Wallis test) or parametric statistic test (Students t-test and ANOVA) to evaluate variable dependencies, respectively.

Ranked parameters with exactly two groups were subjected to Fisher’s exact test, which utilized double-dichotomous contingency (2 × 2) tables. In case at least one of the ranked parameters exceeded the group size of 2, Pearson’s Chi-squared test was applied. Correlations between two metric variables were analyzed by Pearson product–moment correlation coefficient and Spearman’s rank correlation test, respectively.

Various candidate patterns were identified by clustering methods (supervised and unsupervised) or principal component analysis. Gene set enrichment analysis (GSEA) was performed using the WEB-based Gene SeT AnaLysis Toolkit (WebGestalt) website (37). Correlations between a specified variable and the expression of genes in certain signaling pathways were visualized by network maps obtained from the KEGG (Kyoto Encyclopedia of Genes and Genomes) database. Group-based differences were visualized by −log2 fold changes of median or mean counts, depending on their distribution.

OS was calculated using Cox proportional hazard models. Hazard ratios with appropriate 95% confidence intervals, as well as p-values calculated by the Wald test, the score (log-rank) test, and the likelihood ratio test, were used as statistical measures.

To overcome multiple testing variances, p-values were corrected through application of false discovery rate. The cutoff for statistical significance was set at p ≤ 0.05 after adjustment.

## 3 Results

### 3.1 Differential expression of metallothioneins in HGSOCs

**Figure 2:**
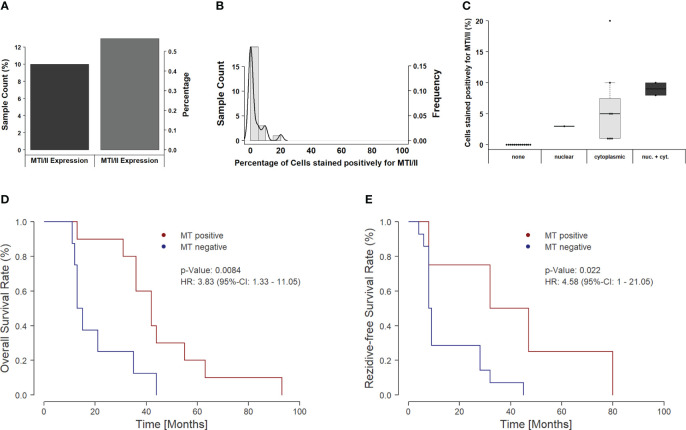
Distribution and clinical impact of MT expression in the overall cohort. **(A)** The figure shows a bar plot of samples with and without MT immunoexpression. Around 60% of all samples do not show a detectable immunoexpression of MTs, whereas 40% show at least one positive-stained tumor cell. **(B)** The figure depicts a combined histogram with density plot. The x-axis shows the percentage of stained cells for each sample, and the y-axis shows the sample count with the given percentage of stained cells. In the majority of the samples, only a few cells are positively stained (up to 10%). Only a minor amount of tumors present up to one-fifth positively stained cells. **(C)** Influence of subcellular localization on staining intensity. If only nuclear expression of MTs is found, then the overall amount of positively stained cells remains below 5%. For cases with cytoplasmic localization of MTs, overall, a higher number of positive cells can be seen; however, the individual values spread over a broad range between 1% and 20%. If both the nuclear and cytoplasmic staining occur, the highest number of stained cells can be found (8%–10%). **(D)** Kaplan–Meier curve for overall survival in dependency of MT expression. Positively stained tumors show a significantly prolonged overall survival (p = 0.0084). The median survival rate increases from 14 months to 42 months, and the 3-year survival rate rises from 12.5% to 80.0%. **(E)** Recurrence-free survival in MT-positive versus MT-negative tumors. Similar to OS, the recurrence-free survival (RFS) increases significantly, if an MT expression can be detected. Median RFS increases from 8.5 months to 39.5 months with an increase in 3-year RFS rate from 7.1% up to 50.0%.

Overall, MT immunoexpression could be detected in about 43% (10/23) of all HGSOC samples (**Figure 2A**). Moreover, those positive samples showed positivity only in a minor proportion of tumor cells, ranging between 5% and 20% (**Figure 2B**).

**Figure 3:**
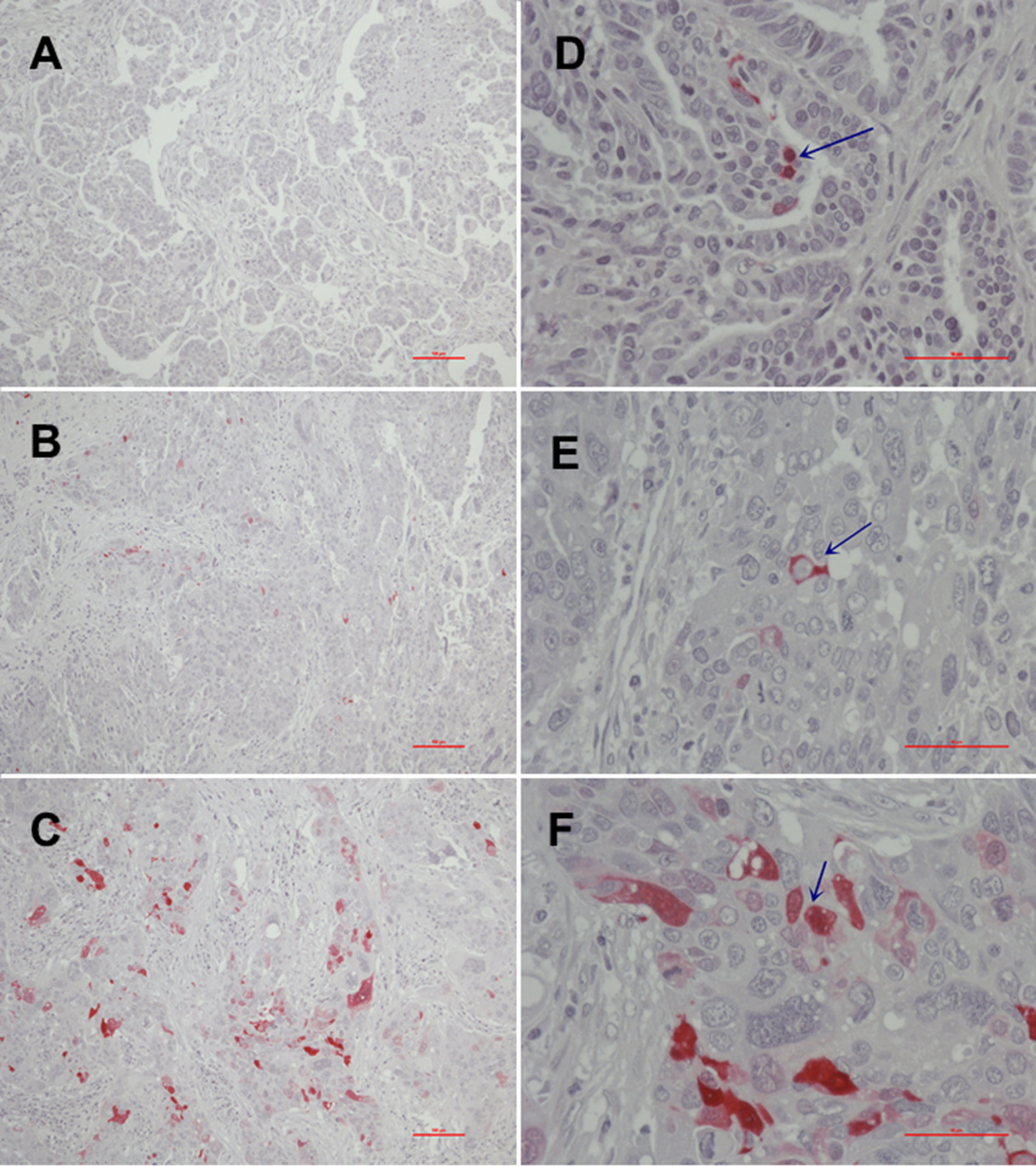
Immunohistochemical MT-I and MT-II expression [**(A)**, no expression (0); **(B)**, moderate to intermediate expression (2+); **(C)**, high expression (3+)] and MT subcellular localization [**(D)**, nuclear; **(E)**, cytoplasmic; **(F)**, nuclear and cytoplasmatic].

In addition to overall positivity, we also analyzed the subcellular distribution of MTs. Of the 10 positive tumor samples, only one sample showed an exclusive nuclear staining pattern, whereas the majority of cases (n = 7) showed cytoplasmic MT expression. Only two samples showed up with both nuclear and cytoplasmic staining. Interestingly, the subcellular localization seems to be of importance for action and activity of MTs. The sample with nuclear-only staining showed only low immunoexpression, whereas immunoexpression rose with the proportion of MT staining from cytoplasmatic to both nuclear and cytoplasmatic (**Figures 2C**, **3**).

### 3.2 Metallothionein immunoexpression is linked with increased overall and progression-free survival in HGSOCs

Overall, the number of positively MT-stained cells shows a significant influence on OS in the recent cohort [likelihood ratio test, p = 0.0262; score (log-rank) test, p = 0.0569; Wald test, p = 0.0478]. In particular, when binarized in expressing/non-expressing cases, the calculated significance further rises [likelihood ratio test, p = 0.0144; score (log-rank) test, p = 0.0129; Wald test, p = 0.0084], showing prolonged survival in positively stained tumors. Not only the five-year survival rate (50.0% vs. 26.3%; odds ratio, 2.66) but also the 3-year survival rate (75.0% vs. 47.4%; odds ratio, 3.17) was notably improved between both groups (**Figure 2D**).

Moreover, progression-free survival (PFS) shows the same association to MT protein expression levels—a shortened PFS in MT-negative samples [likelihood ratio test, p = 0.0222; score (log-rank) test, p = 0.0503; Wald test, p = 0.0345] (**Figure 2E**).

Importantly, subcellular spatial distribution/localization seems to be an important prognostic factor in this context. The present data show that the survival-dependent effects are only based on those cases showing cytoplasmic or combined cytoplasmatic/nuclear immunoexpression of MTs [likelihood ratio test, p = 0.0101; score (log-rank) test, p = 0.0118; Wald test, p = 0.0076].

### 3.3 Metallothionein expression is associated with strongly increased immune cell infiltration

**Figure 4:**
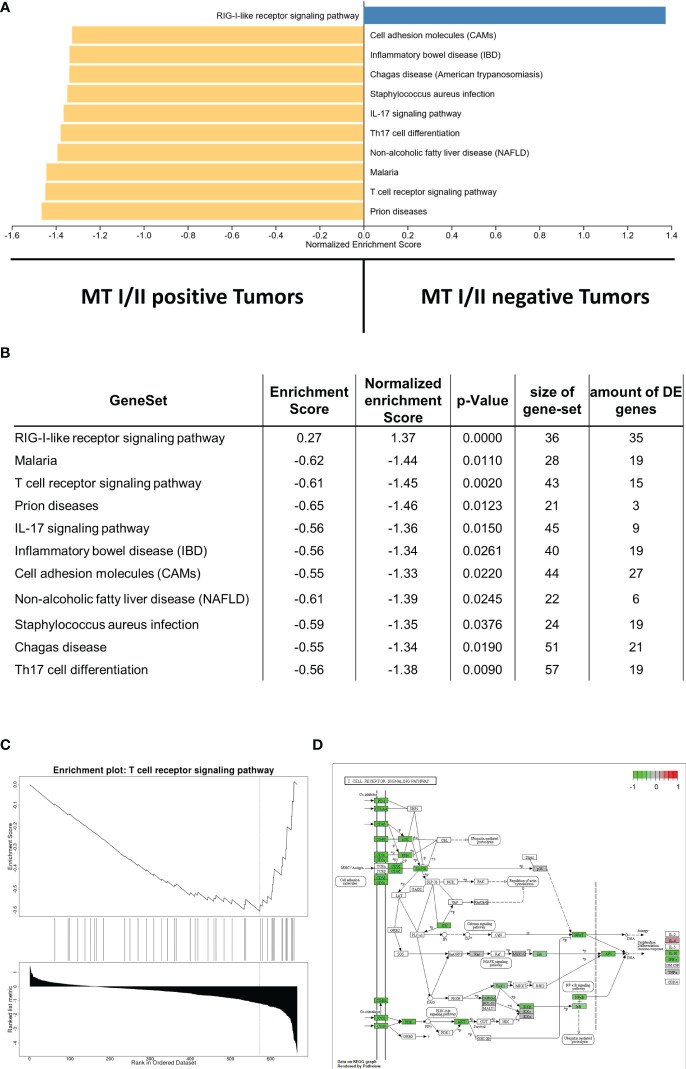
**(A)** Gene set enrichment analysis displaying correlations between MT-I/II abundance and the amount of gene expression in certain signaling pathways. Pathways linked to MT-I/II–negative tumor samples are displayed in blue, whereas pathways linked to MT-I/II-positive tumors are shown in yellow. **(B)** List including the statistical metrics of the enrichment analysis displayed in **(A)**. **(C)** Enrichment plot describing the representation of genes linked to T-cell receptor signaling. Similar to other immune pathways (**Supplementary Figure 1**), a negative enrichment score is shown, indicating a high expression in MT-I/II-positive vs. MT-I/II–negative samples. **(D)** KEGG pathway map displaying genes involved in T-cell receptor signaling. Pathway members highlighted in green show induced gene expression levels in tumor samples with detectable MT overexpression.

In the present cohort, we found high gene expression levels of certain immune response pathways associated with MTs. GSEA revealed that all gene sets significantly enriched in MT-I/I– positive samples comprise immune-associated pathways, including T-cell receptor signaling pathway, T_H_17 cell differentiation, interleukin-17 (IL-17) signaling pathway, or cell-adhesion molecules. An overview of the GSEA is illustrated in **Figure 4A**; a list with the complete statistical metrics is given in **Figure 4B**. Specific enrichment plots for each of the aforementioned pathways are displayed in **Figure 4C** and **Supplementary Figure 1**.

In addition to the pathways refered by the GSEA, we have a specific look on those pathways that are important for an anti-tumor immune response, such as T-cell receptor signaling (**Figures 4C, D**), B-cell receptor signaling (**Supplementary Figure 2A**), NK cell–mediated toxicity (**Supplementary Figure 2B**), leucocyte transendothelial migration pathways (**Supplementary Figure 3A**), and IL-17 signaling (**Supplementary Figures 1B**, **3B**). Furthermore, MT immunoexpression seems to strongly affect phosphoinositide 3-kinases (PI3K) signaling (**Supplementary Figure 4A**).

**Figure 5:**
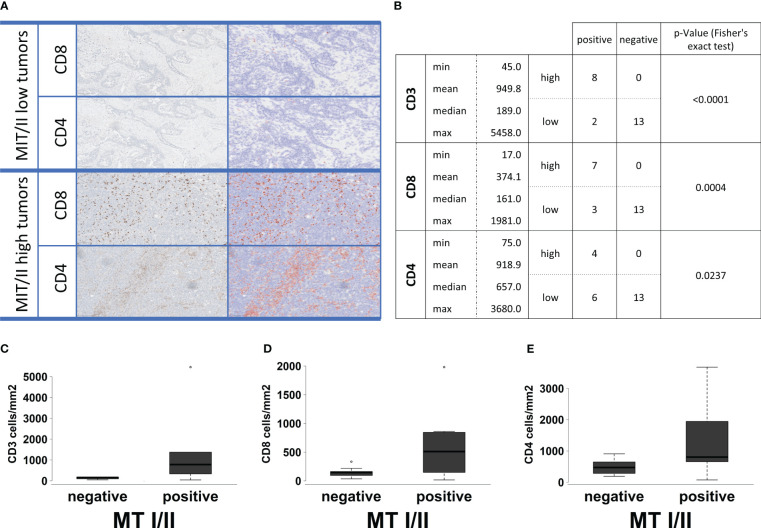
**(A)** Examples for the automatic software-based analysis of immune cell infiltration in our whole cohort of 23 samples separated into MTI/II low (upper two lines) and high (lower two lines) HGSOCs. Each first row represents a staining for CD8-positive T cells, whereas the second row represents a staining for CD4-positive T cells. Cell separation and counting via QuPath leads to blue-shaded negatively and red-shaded positively counted cells. High infiltration levels of T cells could only be observed for MT-I/II rich tumors. **(B)** Basic statistical analysis for infiltration levels with CD3-, CD8-, and CD4-positive cells in MT-I/II–positive and MT-I/II–negative tumors. The p-value was calculated by Fisher’s exact test because both variables display a double-dichotomous correlation. **(C–E)** Correlation between MT-I/II expression (negative and positive) and infiltration (cells/mm^2^) of CD3-**(C)**, CD8- **(D)**, and CD4-positive immune cells **(E)**.

In particular, T-cell receptor signaling (**Figures 4C, D**) shows overexpression of most surface receptor molecules, including co-inhibitory PD1, cytotoxic T-lymphocyte-associated protein 4, and co-stimulatory signals, e.g., inducible T-cell costimulator, CD28, and CD40L. In addition, an upregulation of the different CD3 isoforms as well as CD4 and CD8 can be observed. This could be also shown immunohistochemically, with a high correlation between the respective markers and the infiltration density (cell/mm^2^) of the respective cell type (CD4, p = 0.0004, rho = 0.46; CD8, p < 0.0001, rho = 0.66; **Figure 5A**). On a group level (high vs. low infiltration density defined by mean cells/mm^2^), Fisher’s exact test reveals a significantly higher infiltration with overall T cells (CD3, p < 0.0001), especially not only CTC (CD8, p = 0.0005) but also helper cells (CD4: p = 0.0237), all without exactly computable odds ratios due to the lack of high infiltration samples in the MT-negative section. An overview of the exact distribution metrics can be found in **Figures 5B–E**.

Furthermore, downstream signaling cascades including lymphocyte-specific protein tyrosine kinase, roto-oncogene tyrosine-protein kinase Fyn, zeta-chain-associated protein kinase 70, IL-2–inducible T-cell kinase, nuclear factor of activated T cells and Jun proto-oncogene, AP-1 transcription factor subunit (AP1), as well as resulting IL-10 and interferon-gamma (IFNγ) expression levels generated significantly higher counts in the MT-positive compared with the negative group.

Despite of the high transforming growth factor beta (TGFβ)/IFNγ levels, Suppressor of Mothers Against Decapentaplegic (SMAD2/3) seem to be downregulated in MT expressing samples, indicating no overall activation of canonical TGFβ signaling (**Supplementary Figure 4B**). Contrarily, PI3K signaling pathway as an alternative downstream cascade (a part of the non-canonical TGFβ signaling) showed a strong induction in MT-positive cases, including not only different types of activating surface molecules but also PI3K and Rho family-alpha serine/threonine-protein kinase (AKT1) itself as well as additional different AKT1 downstream targets (**Supplementary Figure 4A**).

## 4 Discussion

MTs prevent damage in normal cells by protecting against oxidative stress, enhancing tissue repair and modulating immune response (17). Nevertheless, MTs can also contribute to a more aggressive phenotype and therapy resistance in cancer cells, resulting in worse prognosis and outcome (17). Although there is an overexpression in breast, uterine, and skin carcinomas, MTs are downregulated in prostate carcinoma/adenoma, small cell lung carcinomas, and hepatic carcinomas (38). Whereas recent studies have shown a tumor suppressive role of some MT isoforms in papillary thyroid carcinoma, cancers of the large intestine, and hepatocellular carcinomas (39–42), MTs have also been shown to promote the progression of adenocarcinoma of the breast and lung (43–45). In melanomas, MT expression has been reported to be associated with abnormal cell growth and malignant behavior (28).

In previous studies, we already published an association between the strength of an anti-tumor immune response and the response to platinum-based chemotherapy in this well-established HGSOC cohort, resulting in a prognostic mRNA-based immune gene signature for prediction of early platin resistance (33). Later on, we investigated the supportive role of other components of the tumor microenvironment, especially cancer-associated fibroblasts, on tumor biology, sensitivity against chemotherapy, and their immunosuppressive role in this cohort (32). Nevertheless, the reasons for varying immune cell infiltration and activity levels remained largely undiscovered. We hereby present the first study combining broad, digital mRNA screening of anti-tumor immune response associated genes and their relation to MTs in ovarian cancer, now elucidating this missing gap.

MT expression has been shown to be associated with the number and activity of immune cells during immune response in breast cancer (46, 47). In cutaneous malignant melanoma, MT overexpression was found to be related to the presence of tumor-infiltrating CD68+ macrophages (28). Dutsch-Wicherek et al. observed an inhibition of the immune response by MTs in pharyngeal squamous cell carcinoma, indicating that MTs are involved in the remodeling of the immunosuppressive tumor microenvironment (48). According to our own results, T cell receptor signaling shows strong activation in MT-positive tumors, indicating an intensified activity of tumor infiltration by T cells. In particular, a strong expression of the different CD3 isoforms as well as CD4 and CD8 has been observed (**Figure 4D**). Furthermore, activated downstream signaling cascades resulting in elevated IFNγ expression in the MT-positive subgroup could be proven. In addition, a higher expression pattern of perforin and several granzymes could be detected, leading to the assumption of acute, targeted, and regulated anti-cancer immune response in MT-positive samples. This activation associated with MT immunoexpression of the cancer cells itself mainly comprises but is not limited to T cells and NK cells. Moreover, a shift in the balance between T helper cells type 1 and 2 (T_H_1 and T_H_2) could be observed.

On the first note, the biological association between MT expression and immunoactivity in cancer may not be obvious. Subramanian Vignesh and Deepe demonstrated the direct immunomodulatory role of MTs as an important component of the innate and adaptive immune systems, thereby influencing a variety of reactions, i.e., redox status, enzyme function, and cell signaling (49).

Although we did not quantify Zn^2+^ levels in our cohort directly, it is highly likely to conclude that MTs regulate the immune system in HGSOC by regulating zinc levels, as MTs are described as the main regulators of intracellular zinc homeostasis (20).

The major problem with zinc metabolism is that both zinc deficiency and excessive amounts of zinc may lead to increased cancer risk (50–52). In addition, inflammatory processes are often linked to alterations in zinc metabolism (20). In particular, patient groups with zinc deficiency have a higher risk in developing sepsis (20). Mouse models displaying zinc deficiency were associated with an impaired inflammation, resulting in increased bacterial burden, organ damage, and increased overall mortality (20, 53, 54). Intracellular zinc signals are required to produce proinflammatory cytokines as IL-1B, IL-6, and tumor necrosis factor (TNF) (20, 55). Higher levels of extracellular Zn^2+^ will also suffice (20, 56, 57). In addition, extracellular Zn^2+^ is involved in recognition of MHC-I on target cells (58, 59). NK cells seem to display less lytic activity during zinc deficiency, most likely due to decreased stimulation from T cells via IL-2 (20, 60). The polarization of CD34+ progenitors toward NK lytic cells was achieved by supplementing zinc *in vitro* (61).

T cell activity, their development and basic functions are implied to be strongly impaired by zinc deficiency (20, 62–64). Suggested molecular mechanisms include the dependency of thymulin on zinc ions as cofactor or altered B-Cell Lymphoma 2 (BCL2) expression levels, resulting in apoptosis of T cell progenitors (20, 62, 63). Moreover, an abundance of Zn^2+^ is essential for maintenance of different T cell subsets (20). Its deficiency leads to reduced assembling of IFNγ, IL-2, or TNFα, which are crucial T_H_1 cytokines. In contrast, the effect is less significant regarding the T_H_2 response (IL-4, IL-6, and IL-10), thereby disrupting their balance (20). Furthermore, zinc deficiency leads to losses of naive B cells; hence, antibody production is decreased (63).

However, it is important to keep in mind that different cancer entities can react differently to changes in zinc homeostasis and MT expression. On the basis of recent studies, the results remain controversial regarding the influence on chemotherapy resistance in lung cancer (65, 66). There may be no effect in primary germ cell tumors (67). In colorectal cancer, MT expression is linked to enhanced chemosensitivity (68, 69).

It remains to be seen in which part MTs contribute to tumor immunology or which other possible explanations are actually relevant *in vivo*.

For ovarian cancer, it has been shown that the presence of TILs positively correlates with survival (70, 71). On the other hand, immunosuppressive factors also play a significant role in EOC. Regulatory T cells have been demonstrated to create an immunosuppressive environment in patients with EOC, resulting in poorer survival (72). In the present study, MTs as mediators of the immune response significantly influence survival in platinum-treated patients. In particular, when binarized as expressing/non-expressing cases, we observed a prolonged OS as well as significantly improved not only 5-year but also 3-year survival rates. Furthermore, PFS shows a similar association with MT protein expression levels, indicating a shortened PFS in MT-negative samples. Importantly, subcellular spatial localization seems to be an important prognostic factor in this context. The present data demonstrate that the prognostic effects are solely dependent on cytoplasmic MT expression.

## Data availability statement

The original contributions presented in the study are included in the article/**Supplementary Material**. Further inquiries can be directed to the corresponding author.

## Ethics statement

The study was approved by the Ethics Committee of the Medical Faculty of the University Duisburg-Essen (protocol no. 16-6916-138 BO). The studies were conducted in accordance with the local legislation and institutional requirements. The human samples used in this study were acquired from primarily isolated as part of your previous study for which ethical approval was obtained. Written informed consent for participation was not required from the participants or the participants’ legal guardians/next of kin in accordance with the national legislation and institutional requirements.

## Author contributions

Conceptualization: EM, MW, and FM. Methodology: EM, FM, and SB. Software: MW and FM. Validation: PB, SB, and FM. Formal analysis: EM, MW, and FM. Investigation: EM, LH, PB, JS, SB, and FM. Resources: FM, and KWS. Data curation: PB, PM, EM, BJ, JS, and FM. Writing—original draft preparation: EM, MW, AB, and FM. Writing—review and editing: EM, PB, SB, LH, PM, MW, JS, RK, BJ, KWS, AB, and FM. Visualization: EM, JS, MW, and FM. Supervision: FM. Project administration: AB and FM. Funding acquisition: FM, RK, and KWS (institutional). All authors contributed to the article and approved the submitted version.
