# Supplementary figures and images for: Tumor cell cytoplasmic metallothionein expression associates with differential tumor immunogenicity and prognostic outcome in high-grade serous ovarian carcinoma
Source: Front Oncol. 2023 Nov 8;13:1252700. doi: 10.3389/fonc.2023.1252700 (PMC10663300; doi:10.3389/fonc.2023.1252700)

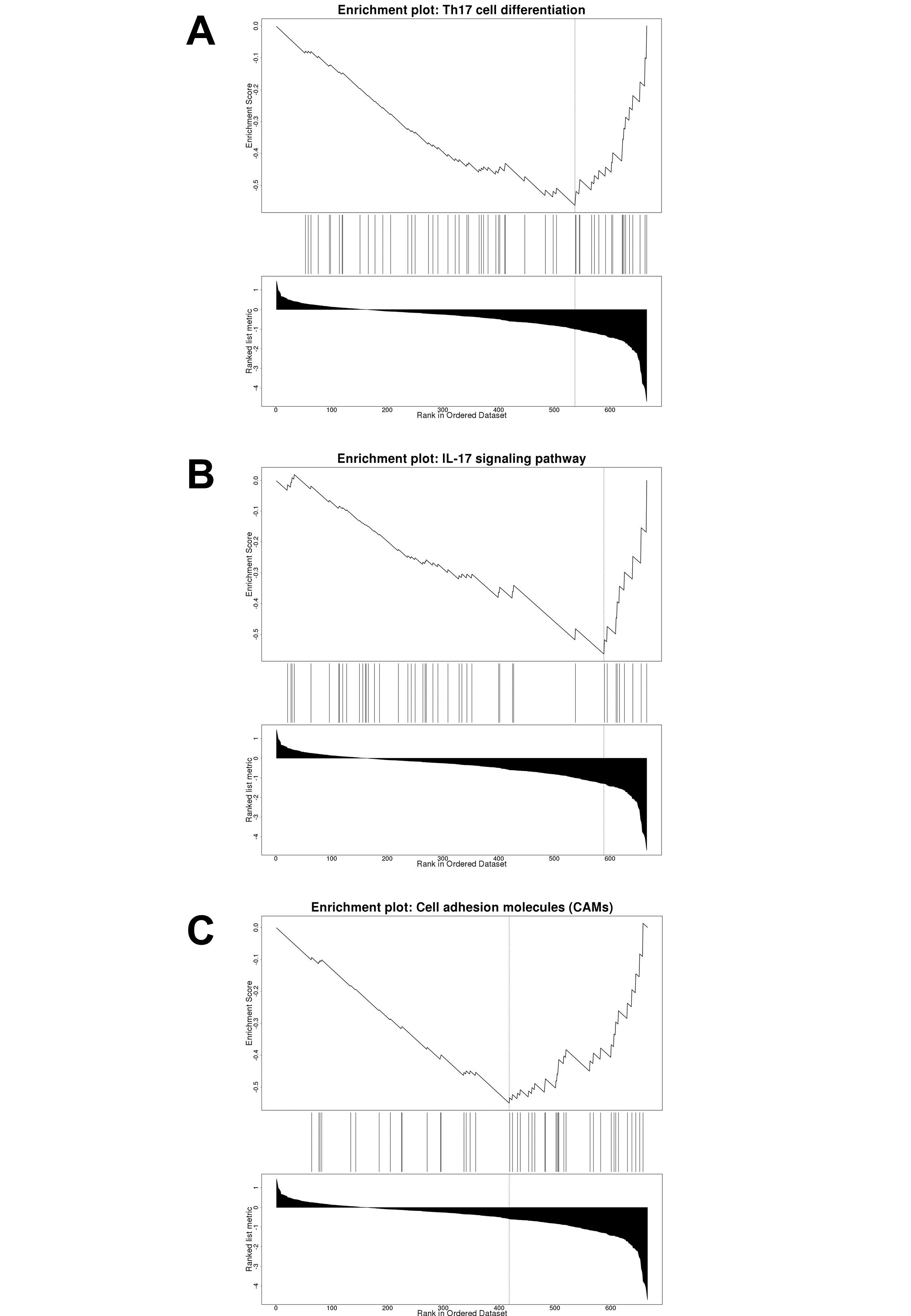

Supplement: Supplementary file 1 [file Image_1.tiff]

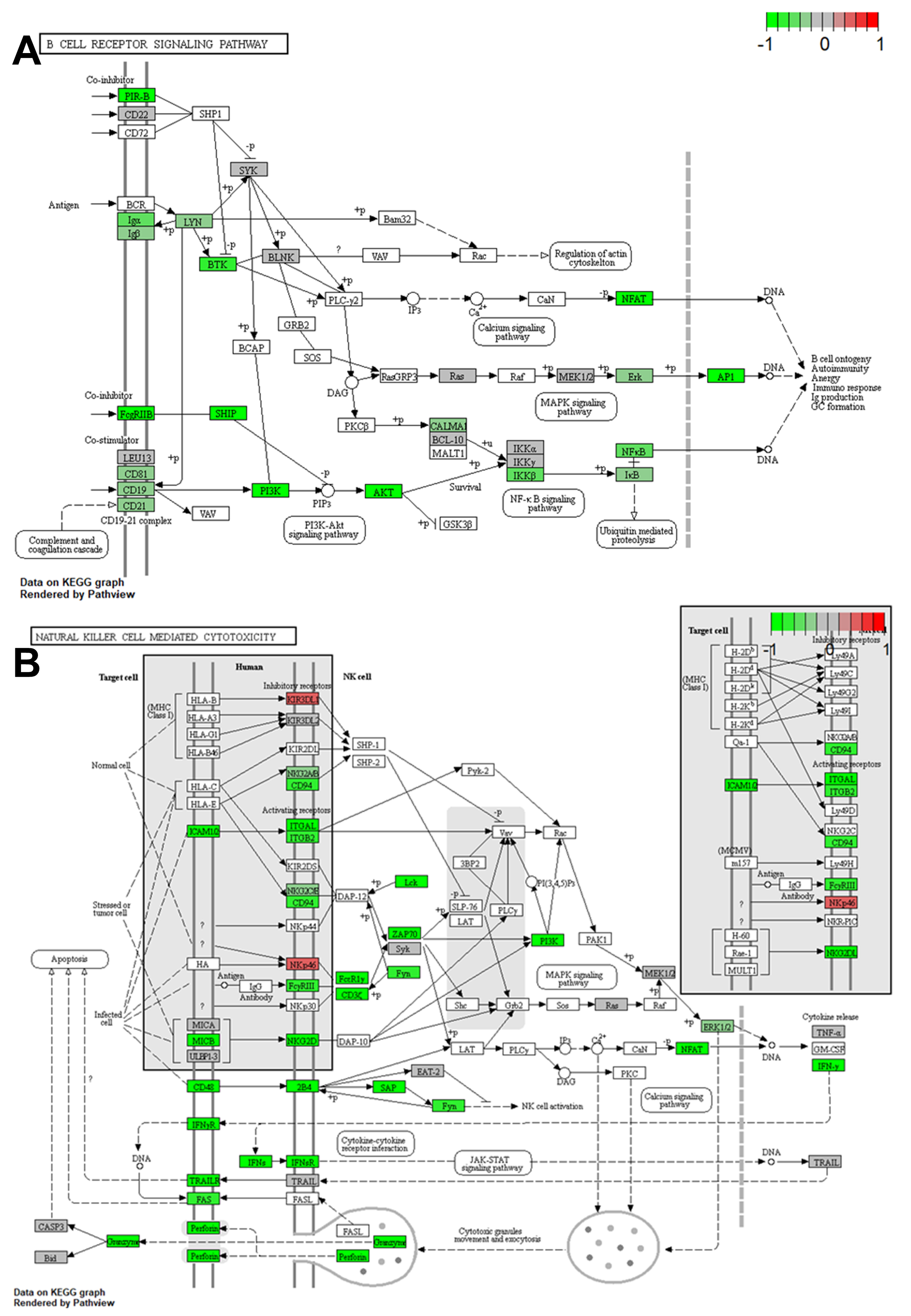

Supplement: Supplementary file 2 [file Image_2.tiff]

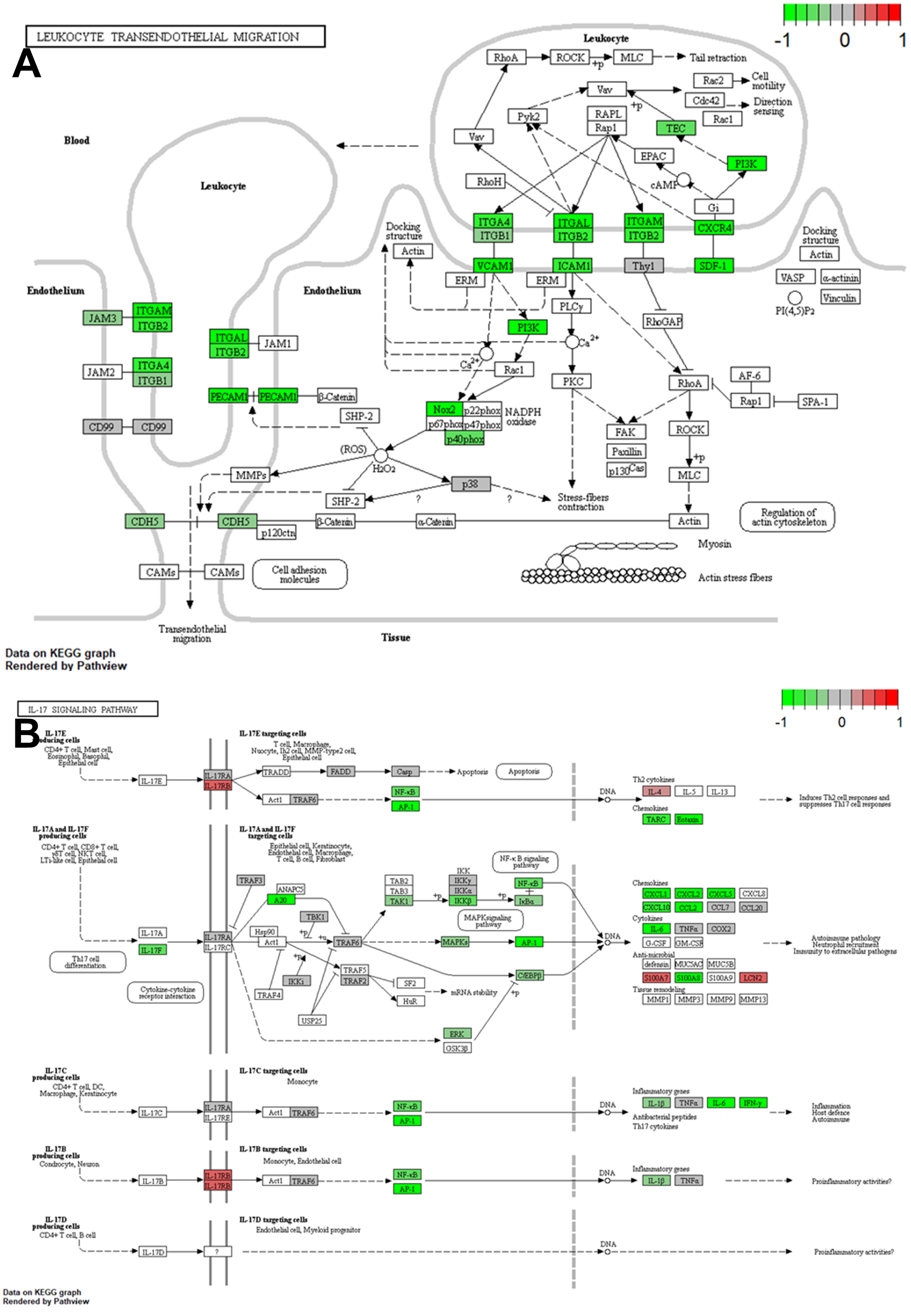

Supplement: Supplementary file 3 [file Image_3.tiff]

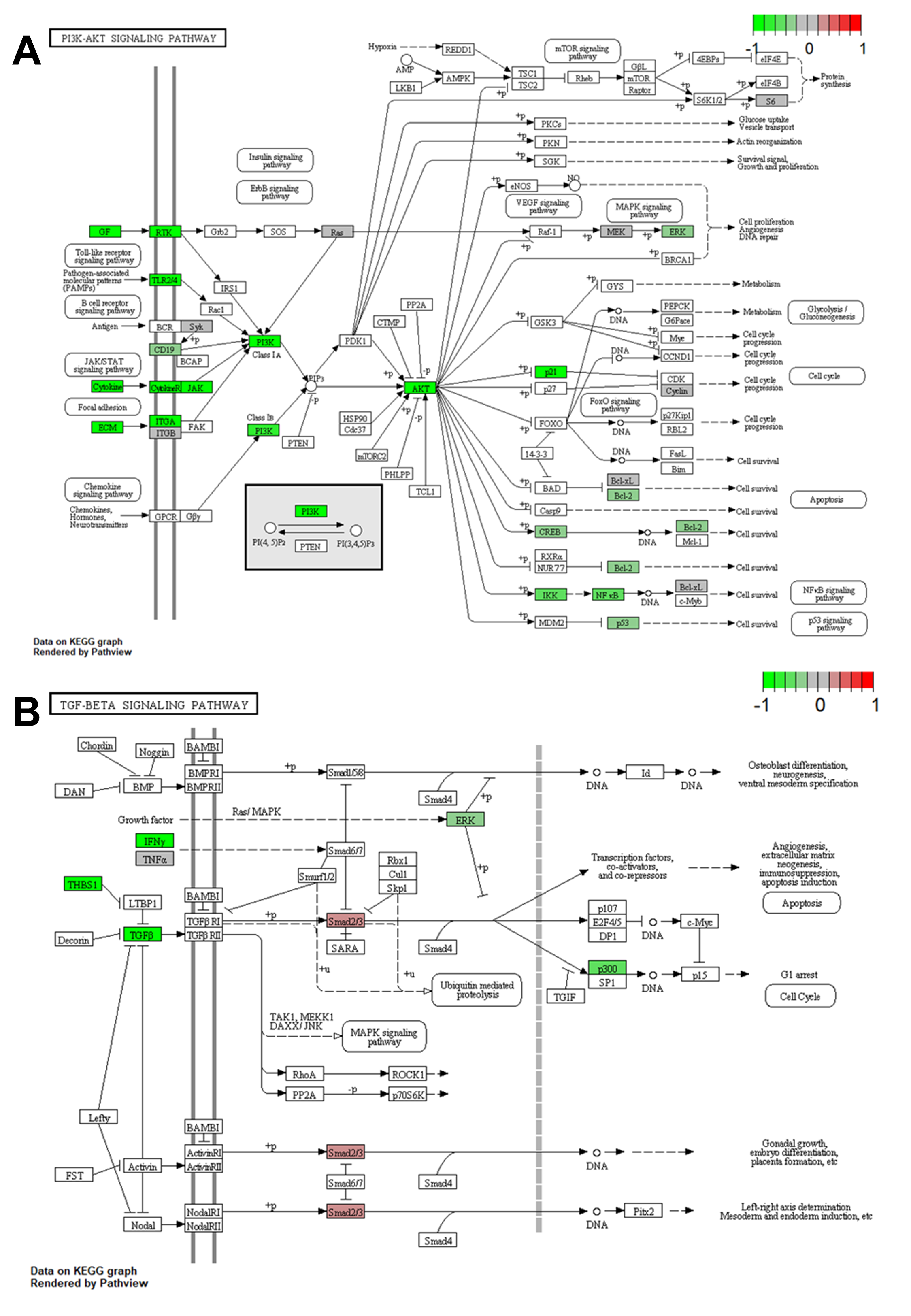

Supplement: Supplementary file 4 [file Image_4.tiff]
